# Building music AI in Nigeria: Korin AI and the contradictions of GenAI futures

**DOI:** 10.1007/s00146-026-03247-8

**Published:** 2026-08-12

**Authors:** Tolúlọpẹ́ Ọ̀kẹ́, Robert Prey, Femke de Rijk

**Affiliations:** 1 University of Groningen, Groningen, Netherlands; 2 University of Oxford, Oxford, UK

**Keywords:** GenAI, Nigerian music, Korin AI, Africanfuturism, Decolonial AI, Music AI, Global South, Coloniality/modernity

## Abstract

Most accounts of the intersection of AI and culture begin in the Global North and, despite the intensity of current debates and a growing body of scholarship, rarely move beyond its infrastructures, policies, implications, and aesthetics to examine how AI’s futures are being negotiated elsewhere. This geopolitical imbalance obscures how creative producers in other regions encounter and reinterpret AI within distinct socio-cultural and infrastructural realities, rendering artists in the Global South as passive adopters of technologies from “elsewhere.” We address this imbalance by centering Nigeria, particularly its music industry, as a site for understanding how generative AI (GenAI) is contested and localized. More specifically, we examine Korin AI, a Nigerian-owned AI music generation platform, to analyze how AI futures in Nigerian music are imagined, negotiated, and materially organized within conditions of infrastructural scarcity, legal ambiguity, and global coloniality. Based on ethnographic fieldwork in Lagos, including interviews with musicians, producers, platform founders, and entertainment lawyers, we argue that Korin AI is best understood neither as a fully realized decolonial alternative nor simply as a case of technological dependency, but as a contradictory and compromised project of future-making. On one level, it embodies Africanfuturist and decolonial aspiration by foregrounding African musical data, languages, and genres, and by insisting that African sounds should not remain absent from AI’s musical futures. On another, it remains tethered to unequal systems of data, computation, law, and capital, reproducing new internal hierarchies even as it seeks to resist external ones. Korin AI thus reveals what it means to build music AI in Nigeria from within, rather than outside, the enduring structures of modernity/coloniality.

## Introduction

Most accounts of the intersection of AI and culture begin in the Global North and, despite the intensity of debates and a growing body of scholarship, rarely move beyond its infrastructures, policies, implications, and aesthetics to examine how AI’s futures are being negotiated elsewhere (Jaramillo-Dent and Arora 2025; Arora and Natale 2025; Natale et al. 2025). This geopolitical imbalance obscures how creative producers in other regions encounter AI within their distinct socio-cultural and infrastructural realities. In the process, AI appears as a technology that originates from “elsewhere,” with artists and cultural workers in the Global South rendered as passive adopters.

In this study, we address that imbalance by centering Nigeria, particularly its music industry, as a site for understanding how generative AI (GenAI) is contested and localized. More specifically, we address the central analytical problem of how AI futures in Nigerian music are imagined, negotiated, and materially organized within conditions of infrastructural scarcity, legal ambiguity, and global coloniality. Moving beyond the framing of GenAI as another technological import, we investigate how Nigerian actors localize its meanings, uses, and infrastructures, and the contradictions that manifest in the process.

Drawing on Africanfuturist and Decolonial AI perspectives, we position actors in the Nigerian music sector as active re-interpreters who situate AI within local epistemologies, material constraints, and cultural aspirations. In doing so, we reveal how their engagements with GenAI generate alternative imaginaries of technological belonging and creative labor beyond dominant Global North paradigms, even as these imaginaries remain tethered to external infrastructures and uneven power relations. We therefore argue that Nigerian engagements with GenAI are best understood as contested efforts to reorganize musical futures from within, rather than outside, the enduring structures of modernity/coloniality. In the sections that follow, we review the development of Nigerian popular music and then provide an overview of AI in music and Nigeria, setting the context for our case study.

## Nigerian music

Nigeria’s vibrant and heterogeneous music scene has often been described as central to Africa’s contemporary soundscape, as “the musical heartbeat of Africa” (Servant 2003). With a population of about 230 million in 2024, the country is the continent’s most populous nation and one of its largest cultural economies. Although other African music markets, especially South Africa and Kenya, offer important points of comparison, Nigeria occupies a particularly significant place because of the scale, visibility, and circulation of its music industry.

Nigerian artists have had a profound impact on musical culture across the continent and globally. The country’s music industry, renowned for Afrobeats as well as Jùjú, Fújì, Highlife, and other popular traditions, has become a major force in Africa’s wider entertainment economy. PwC’s recent Africa entertainment and media outlook identifies Nigeria as the fastest-growing entertainment and media market in the region, recording 11.2% growth in 2024 and outpacing comparable markets, such as Kenya and South Africa (PwC 2025). At the same time, IFPI (2025) reports that Sub-Saharan Africa’s recorded music revenues grew by 22.6% in 2024, with the region surpassing US$100 million for the first time. These figures foregrounds why Nigeria offers a critical vantage point for understanding creative production outside the Global North.

Historically, Nigerian popular music has evolved through hybrid forms that merge indigenous traditions and transnational influences. Okafor and Okafor (2009, pp. 149–52) categorize Nigerian music into three broad streams: (A) derivatives of traditional music (such as Yorùbá’s Apala and Sakara, Ígbò’s Akuko n’egwu, and Hausa’s Gwoje); (B) forms that reflect interaction between local and Western cultures (e.g., Highlife, Jùjú, Fújì); and (C) imported or diasporic forms (e.g., Reggae, Hip Hop, Ozziddi). This categorization highlights how Nigerian popular music is both adaptive and resistant, constantly negotiating colonial inheritances and global market pressures.

Highlife, for example, developed through the circulation and recombination of brass-band music, palm-wine guitar styles, jazz idioms, European instruments, and West African dance forms, before becoming deeply embedded in urban popular culture across West Africa, including Nigeria (Collins 1989). Jùjú music similarly emerged through the confluence of Yorùbá vocal and percussion traditions, Christian congregational singing, and African and Western popular styles, eventually reaching international audiences through figures such as King Sunny Adé (Waterman 1990). This longer history of syncretic adaptation continued with Afrobeat, which Fela Kuti formulated by bringing Yorùbá musical traditions into conversation with highlife, jazz, funk, blues, and a radical politics of dissent. In Afrobeat, the sonic resources of Black Atlantic modernity were reworked into a distinctly Nigerian form through which anti-colonial critique, urban frustration, and political resistance could be voiced musically and ideologically (Olaniyan 2004; Veal 2000).

Just as importantly, this work of cultural translation has also involved technology. Nigerian musicians and industry actors have repeatedly appropriated global media technologies as local instruments of innovation, access, and democratization. As Nkechi (2012, pp. 65–68) observes, the advent of hip-hop and youth-driven popular forms in the 1990s marked a democratization of Nigerian musical production where “youth discovered in an easy-to-manipulate technology a means of production that liberates potential musicians from former hegemonic apprenticeship.” The subsequent spread of computers and software such as FL Studio (formerly FruityLoops) made production even less expensive and less studio bound. A similar pattern marked the arrival of digital distribution. Nigerian artists now bypass record labels through streaming platforms and viral content strategies (Adedeji 2023, p. 133). The social media logic of visibility, articulated in the Nigerian vernacular as the desire to “blow,” has redefined artistic success, incentivizing virality and encouraged participatory trends such as the “Buga Dance Challenge” (Adedeji 2023, pp. 139–41). As Onyekwelu and Sylvanus (2024) note, digital distribution has disrupted record-label oligopolies, placing monetization and control more directly in artists’ hands.

This digital transformation also catalyzed a shift in Nigerian music’s aesthetic hybridity. Genres now operate across temporal, spatial, and stylistic registers, combining local rhythms and global sonic sensibilities. This continuously evolving hybridity enables Nigerian musicians to “strategically appropriate elements of Western and African cultures into a new musical brand that is neither hegemonic nor nativist” (Obijiaku 2022, p. 31). Afrobeats, now the nation’s most visible musical export, embodies this negotiation. It merges Nigeria’s diverse percussive logics with global pop production; drawing on hip-hop, R&B, Dancehall, House, Amapiano, Highlife, Afropop, and digital production cultures, but often through Nigerian idioms, Lagos-centered industry practices, and diasporic networks (Eze 2020; Serres 2025). The result is a sound that has been embraced transnationally while asserting African subjectivity. As captured by the popular social media hashtag #africatotheworld, Nigerian artists are reclaiming their cultural identities and exporting them globally (Serres 2023). Burna Boy’s *African Giant* (2019) exemplifies this transnational aesthetics of pride, blending Pan-Africanist themes with diasporic rhythm and critique (Aruku et al. 2025).

This success has occurred despite significant challenges. The music industry in Nigeria has long suffered from structural problems, weak copyright enforcement, and infrastructural precarity (Adedeji 2016). Since the introduction of cassette technology in the 1970s, Nigeria’s music sector has been heavily shaped by piracy or what Simmert (2020) describes as “informal music distribution.” The inability to generate sustainable profits led major Western labels to withdraw from the country in the early 1990s, and most domestic record labels soon collapsed (ibid., p. 334). In the vacuum left by the disintegration of formal distribution, “music marketers” based in Lagos’s Alaba International Market, the country’s largest electronics hub, emerged as key intermediaries, controlling much of the circulation of recorded music (Adedeji 2016). Within this context, Nigerian recording artists have constructed careers despite a fragile, and at times virtually non-existent, recording industry infrastructure.

In recent years, major record labels such as Sony and Universal have moved back to Nigeria, as they attempt to capitalize on the global popularity of the Afrobeats genre. Nevertheless, this success creates its own problems. The contemporary Afrobeats economy is increasingly entangled with global labels, distributors, platforms, and data infrastructures. Recent reporting indicates that three foreign music distribution companies, including Empire, Universal Music Group, and Sony Music, accounted for 68% of Nigeria’s total streaming volume in 2025 (Daba Finance 2026). The global enthusiasm for Afrobeats risks commodifying authenticity as multinational labels and platforms push local Nigerian sounds to conform to Western norms (Hesmondhalgh 2013). Algorithmic recommendation systems further intensify this homogenization, privileging algorithm-friendly beats and English-language lyrics over indigenous languages or experimental sounds. As Birhane (2020) warns, these dynamics reflect “algorithmic colonization,” where Global North data regimes impose epistemic and aesthetic dominance through corporate infrastructures.

Streaming and social media platforms based outside of Nigeria have been crucial to Afrobeats’ international growth: *Music in Africa* reports that global streams of Afrobeats grew by 550% between 2017 and 2023, while also noting that the US and UK now stream Afrobeats more than Nigeria does (Hiller 2025). This reveals a structural asymmetry: the music may be Nigerian, but the infrastructures through which it is discovered, distributed, measured, and monetized are often controlled elsewhere. The acquisition of a majority stake in Lagos-based Mavin Global by Universal Music Group is emblematic of this shift as it signals Afrobeats’ commercial value as well as the increasing incorporation of Nigerian labels into Western corporate music structures (Tencer 2024). *Le Monde* describes the dependency problem even more starkly, reporting that despite Afrobeats’ global success, Nigeria’s music industry remains weakly structured and poorly regulated, with many major artists signed and managed through foreign labels and agencies. One industry figure quoted in the article argues that “a lot of money leaves Nigeria” and that neither the industry nor the country benefits as much as it should (Douet 2024).

GenAI thus arrives at an interesting time. Nigerian music producers and artists have historically adapted both global genres and technologies to suit local needs. This history provides an important frame for thinking about music AI in Nigeria. If Nigerian popular music has succeeded by translating global musical genres and technologies into locally meaningful cultural forms, can Nigerians do the same with GenAI? The question is not simply whether Nigerian AI music start-ups can participate in the global GenAI economy, but whether they can translate the cultural success of Nigerian music into technological form: building tools, datasets, interfaces, and business models that remain accountable to Nigerian musical knowledge while still circulating internationally. We will turn to an examination of the challenges and contradictions after first providing some background on GenAI in the field of music.

## GenAI and Nigerian music

“Music AI” encompasses systems that apply AI to composition, production, performance, analysis, and understanding of musical structures (Wei et al. 2025; Wilson et al. 2025). Contemporary generative (GenAI) systems, such as Suno, Udio, MusicGen, Stable Audio, and AIVA, can now produce entire tracks from text prompts, while voice-synthesis technologies enable vocal cloning. Deep learning models, such as RNNs and GANs, have also advanced AI composition significantly (Wang 2025). Li (2024) distinguishes AI from traditional algorithmic composition by emphasizing the former’s capacity to autonomously learn musical structure from data rather than rely on pre-coded rules.

Proponents argue that GenAI opens tangible creative and economic possibilities. As co-creative media, these tools scale cycles of ideation and feedback, assist with melody and harmony exploration, and support mutable musical ecosystems beyond fixed recordings (Singh et al. 2024). Likewise, there are emerging commercial explorations. For example, beyond conventional royalties from streaming or performance, artists can now license not only their musical compositions but also performative signatures like their voice, thus multiplying revenue streams (Wei et al. 2025; Lee 2024; Wang 2025; Epstein et al. 2023). Similarly, producers could distribute stems or algorithmically generated beats as licensable material, multiplying their sources of revenue in ways that extend far beyond the traditional album economy.

More recent developments indicate that the governance and commercial organization of GenAI music are already beginning to be formalized. Major recording labels have started negotiating AI music rights with GenAI start-ups such as Suno and Udio (Darla 2026). YouTube has engaged in discussions with major record companies to secure licenses for music used in AI training, while Meta has recently expanded its collaboration with Universal Music Group (Wei et al. 2025, p. 18). At the level of artistic practice, musicians such as Grimes have already experimented with allowing the public to generate AI music using clones of her voice in exchange for shared royalties (Lee 2024, p. 218). Market analyses project rapid growth in the GenAI music sector, with increasing adoption across composition, engineering, and promotional workflows (Goldmedia 2024a, b; CISAC and PMP Strategy 2024).

GenAI has also been praised for lowering barriers to entry. Just as DAWs consolidated recording, mixing, and composition into accessible software (Chen et al. 2024; Bai 2025), GenAI allows non-specialists to realize complex and experimental ideas, without the need for expensive and exclusive recording equipment. In this regard, it democratizes and functions as an infrastructure for resource-constrained creators. The development of these tools offers greater flexibility and creative autonomy, potentially enabling more equitable participation in the global music economy.

However, these opportunities also introduce new risks. First, there are concerns over authorship, intellectual property, and appropriation. As GenAI models are trained in copyrighted corpora, questions of consent, credit, and remuneration are raised (Hazucha 2022; Goldmedia 2024a, b). In the Global South, where different manifestations of technological advancement have been deployed for colonial and techno-capitalist exploitations, there is a growing concern of cultural appropriation and decontextualization when GenAI intersects with indigenous cultures and their repertoire of music. As Dugeri puts it, AI systems extract “traditional cultural expressions (TCEs) from their cultural contexts, transforming them into commodities without acknowledgment or compensation to the original communities” (2024, p. 23)**.** Second, there are persistent concerns that AI-generated music lacks expressive and emotional depth. While GenAI outputs demonstrate harmonic coherence, they lack the embodied intentionality, personal experience and cultural resonance that characterize human creativity and intrinsic to human-driven music creation (Gera 2025; Heigl 2025; Spennemann 2024; Wei et al. 2025; Li 2024). Third, AI may exacerbate labor issues and income distortion in the music sector. Projections indicate substantial revenue risks for creators in the absence of compensatory governance (Goldmedia 2024a, b; CISAC and PMP Strategy 2024), while increasing the capital income of those who own and control these technologies, thereby further intensifying inequalities (Cazzaniga et al. 2024; Tatar et al. 2024). Contributing to this economic and labor challenge is the capacity of AI-generated music to produce an unlimited number of derivatives from a finite corpus of originals, thus outcompeting and undercutting the work of musicians.

The fourth risk is the challenge of homogenization, which is particularly concerning when we consider the implications of AI for music in the Global South. As Mehta et al. (2024, pp. 2–5) demonstrate in their study of over 1 million hours of audio datasets used in AI music generation, 86% of the music datasets and 93% of the related research concentrate on the Global North, leaving African repertoires with a measly 0.2% representation.^1^ This is despite the fact that the Global South, and particularly Africa, accounts for a significant proportion of global musical traditions, resulting in non-representation, misrepresentation, cultural homogenization or erasure, and the reproduction of Western harmonic norms. As a result, a generated composition intended to resemble Nigerian Fújì music may sound noticeably off.^2^ These dynamics further increase existing North–South inequality in cultural production, technological ownership, and access to regulatory protections.

For Nigerian musicians, the challenge is in harnessing generative and algorithmic tools for creative expansion while resisting the homogenizing pressures embedded in the very technologies that enable global visibility. Okafor and Okunbor (2024, p. 315) emphasize that optimal AI adoption in Nigeria must recognize the country’s “language diversities, rhythmic structures, melodic structures, harmonic structures, and instrumentation of various ethnic groups.” Similarly, Opuowei (2025) envisions AI as a tool for cultural preservation, enabling digital platforms to showcase performances from diverse Nigerian ethnicities and promote intercultural understanding. Such participatory frameworks resonate with Inie et al. (2023, p. 1) call to “understand the needs of people and communities affected” by AI design. In the Nigerian music context, our findings suggest that this need is articulated less through a desire for full automation than through more grounded forms of augmentation, such as improved production quality, faster prototyping, multilingual songwriting, vocal experimentation, easier access to instrumental generation, the preservation of endangered musical traditions, and expanded possibilities for artists constrained by the cost of conventional studio production.

A recent global survey we conducted (de Rijk and Prey, (2026), p. 64) indicates that Nigerian musicians in the sample were comparatively more positive about, and more likely to experiment with, AI than respondents in the other countries included in the study. These developments mark a shift from AI as back-end optimization to AI as front-stage creativity and authorship. A distinctly Nigerian wave of creative–technical experimentation is emerging. In 2023, Eclipse Nkasi released *Infinite Echoes*, which is well documented and widely described as the first African album co-created with GenAI, and introduced Mya Blue, a Nigerian AI music artist whose early experimentation reworked Victor Uwaifo’s “Joromi.” Around the same period, Ope Brown debuted Mugu Prince (an AI artist, with tracks on Spotify, including the album *Omegapro Scamdemic*), and Obanua’s Entertainment’s Kumi Bora, an AI artist, released the debut album *Discovery*. On the platform side, “Korin AI” (which we will discuss in detail below) is being positioned as the first African-trained GenAI music generator (“kórin” meaning “to sing” in Yorùbá). These initiatives are accompanied by a widening managerial and infrastructural interest in AI, as Nigerian music industry executives (e.g., Excel Joab, Akachi Ígbòko) have publicly advocated for even deeper AI exploration.

However, as Itanyi (2024) observes, Nigeria currently lacks explicit legal frameworks for AI-generated works. This regulatory vacuum compounds existing tensions over copyright, royalties, and authorship that predate AI (Stephen 2011). The result is an uneven ground in which artists navigate both informal creative economies and formal legal absences, relying on improvisation as a mode of governance. The stakes of this negotiation are high. Nigerian music, and particularly Afrobeat, has historically served as a platform for youth liberation and political critique, functioning as a space for self-determination against structural inequality. The integration of AI could potentially either amplify or constrain this role for music within the Nigerian civil society. On the one hand, AI could democratize production and global visibility; on the other, it could deepen dependence on foreign infrastructures that reproduce colonial hierarchies of data and value. In this context, GenAI thus represents both a continuation of Nigeria’s long-standing negotiation with technology and a new ground for asserting decolonial and Africanfuturist imaginaries.

After detailing this study’s theoretical frameworks and our methodology, we will turn to an examination of these imaginaries and manifestation.

## Africanfuturism and decolonial AI

This study adopts an interpretive stance grounded in Africanfuturist and decolonial epistemologies, both of which resist universalist accounts of technology and instead foreground situated knowledge, plural futures, and the right of historically marginalized communities to narrate and design their own worlds. Rather than approaching GenAI as a neutral global infrastructure, we treat it as a contested terrain in which power, culture, and futurity are continuously negotiated. Both Africanfuturism and decoloniality are concerned with attempts to “reshape reality [and futures] on their terms” (Krishnan et al. 2022), but they do so from different temporal orientations. Africanfuturism works by envisioning alternative African futures in the present, while decoloniality wrestles with the enduring past in the present, that is, with the afterlives of colonial power that continue to structure what can be imagined, built, and sustained.

Reality, however, is stubbornly persistent. It repeatedly interjects into and interrupts attempts to imagine and actualize alternatives. Colonial legacies endure in the languages used or ignored, in the uneven distribution of cloud infrastructures and broadband, in legal frameworks that lag behind new forms of exploitation, and in training datasets that subtly marginalize Africa. As Omeje (2015) observes, coloniality is regenerative, carrying “a potential energy to outlive and reproduce itself in perpetuity.” This friction between imaginative and material interventions, between the desire to build otherwise and the persistence of unequal infrastructures, shapes our encounter with GenAI in this study, and necessitates our theoretical framing.

Coined in 2019 by the Nigerian American speculative writer Nnedi Okorafor, Africanfuturism is both a reaction to and a divergence from Afrofuturism, which has often been critiqued for centering diasporic and Western locations at the expense of continental African realities (Okorafor 2019). For Okorafor, Africanfuturism is “concerned with visions of the future,” but it is rooted first and foremost in Africa and in futures imagined from African realities. Although her formulation emerges from speculative fiction, subsequent scholarship has expanded Africanfuturism into a broader onto-epistemic mode of African futures-making that exceeds literary expression (Oke 2025; Cleveland 2024; Bello 2021). In this wider sense, Africanfuturism is not only a matter of representation but of intervention: it names artistic, social, and technological practices that seek to script African futures from within the conditions of the present.

Decolonial AI extends the broader decolonial project into the domains of data and computation. Decoloniality, as articulated by scholars such as Walter Mignolo and Sabelo Ndlovu-Gatsheni, is not simply a critique of colonialism but an ongoing struggle against coloniality, the enduring patterns of power, extraction, and epistemic hierarchy that persist long after formal empires have ended. With AI, these dynamics appear in extractive data regimes, asymmetrical control over infrastructures, and models trained predominantly on Western cultural corpora while African music, languages, and aesthetics remain marginal, distorted, or tokenized. The AI Decolonial Manyfesto captures this succinctly: “AI coloniality extends beyond data colonialism: [it] implies material extractivism, and the use of AI has the capacity to shape reality… [therefore] in insisting on a decolonial AI, we stand for the right of each historically marginalized community to reshape reality on their terms” (Krishnan et al. 2022). This insistence on reshaping reality is crucial. Unlike some strands of postcolonial critique that remain primarily at the level of representation, decoloniality emphasizes praxis, the building of alternatives, infrastructures, and institutions that materially disrupt colonial patterns.

These frameworks allow us to approach Nigerian GenAI music through questions of futurity, sovereignty, survival, and infrastructural dependence. Africanfuturism and Decolonial AI enable us to examine Nigerian GenAI music as a contradictory and compromised project of future-making. The former helps us identify how actors such as Korin AI’s founders imagine and build alternative AI-mediated musical futures from within Nigeria; the latter shows how those futures remain tethered to unequal systems of data, computation, law, and capital. Thus, Korin AI is significant because it reveals what building alternative music-AI futures from within coloniality entails. These contradictions are not incidental to the case; they are constitutive of it. 

## Methods

The research adopts a qualitative, case-study design focused on the city of Lagos, Nigeria, the largest hub of contemporary Nigerian popular music production. The city’s dense networks of studios, record labels, distribution platforms, and informal creative economies make it an ideal place for examining how GenAI is being taken up, contested, and locally reworked. The fieldwork combined semi-structured interviews with ethnographic observation in the Lagos music and tech scene and was conducted in November 2025 and April/May 2026.

Interviews were conducted using purposive and snowball sampling with 25 participants, including musicians, producers, label executives, entertainment lawyers, and tech entrepreneurs. While this number is relatively small, the study is not intended as a statistically representative account of the Nigerian music industry. Rather, it is a qualitative inquiry centered on a particularly rich and emergent case, Korin AI, with the additional interviews providing industrial, legal, and cultural contexts for interpreting that case. Interviews, which lasted between 30 and 120 min, were conducted mainly in English, recorded with informed consent, and transcribed verbatim. Questions explored access to and experimentation with AI tools, attitudes toward automation and creativity, perceived risks and opportunities, and the ethical, legal, and cultural implications of AI-mediated music production in Nigeria. The aim was to build a grounded account of how GenAI intersects with existing infrastructural, cultural, and economic conditions in Nigeria.

The analysis presented here is grounded primarily in the Lagos fieldwork, but it also takes cues from a 5-year global project that includes related surveys and separate interviews conducted by project members. This provides additional comparative and contextual insight into musicians’ platform and AI practices. Most notably, we include broader cross-national findings on musicians’ engagement with AI which helps situate the Nigerian case within a larger comparative frame without displacing the study’s ethnographic focus on Korin AI and its developers.

The fieldwork in Lagos was conducted by the first author, and the ethnographic observations are therefore narrated from his perspective. In the findings and discussion, we move between his situated narration and our joint analytic exposition. Where interlocutors are identified by name in the article, this reflects their explicit consent to be named; other participants were anonymized where appropriate. We acknowledge our dual positionalities as both insiders, culturally and linguistically situated within Nigerian artistic networks, and academic observers embedded in transnational scholarly infrastructures. Following work on insider–outsider positionality, collaborative ethnography and decolonizing qualitative research, we do not treat these field interactions as straightforward processes of extraction, but as relational, dialogical co-productions of knowledge shaped by trust and collaborative reflection (Mullings 1999; Dwyer and Buckle 2009; Thambinathan and Kinsella 2021).

## Findings and discussions: Nigerian music, generative AI, and the case of Korin AI

### Situating Korin AI in the (Pr)axes of decoloniality and African futures building

Lagos could be Nigeria folded into a 3577 km^2^ landmass. In Lagos, life exists at the precipice of uncertainty and polarity. The city fuses chaos and aspiration; it is why the Nigerian poet Olajide Salawu exalts it as a “watershed dream,” a place where everything is possible, but nothing is guaranteed. The “Centre of Excellence,” as Lagos is referred to, offers this dream to all, but it does not withhold its precarity: the city, as Salawu (2021) writes, “does not say/you should not dream of water,/if your arms are lifejackets.” On the morning of my meeting with Philip Olajide-Philips at the University of Lagos in Akoka, Yaba, I moved through those tiny veins of the city that are not only its cluttered river bodies, as Olajide Salawu imagines, but also the tortuous hours spent in heat and “go-slow,” with impatient drivers and passengers navigating around the inadequate but improving road infrastructures of sub-Saharan Africa’s most populous city.

 Philip is one of the co-founders and the CEO of Korin AI, which bills itself as “Africa's first AI Music Generator.” I met Philip at the 5th International Conference on Artificial Intelligence and Robotics. The conference themes, “building responsible AI for Africa” and “domesticating AI for inclusion,” formed a potent backdrop for our conversation. Also noticeable was how the venue captured, in miniature, the contradictions of Lagos and of Nigeria more broadly: the co-existence of excellence and structural inadequacy. Some sections of the hall were bright and carefully arranged, while other corners only received an occasional flicker from unstable ceiling lights. At an event meant to spur productive dialog, the loud music blasting from speakers in the corners of the hall was itself a constraint. At the entrance, a pleasant conference usher tried unsuccessfully to confirm my registration due to an internet outage. I stood there, waiting for a reconnection that never quite came, until my host arrived to escort me in. The scene symbolized something of the wider landscape in which Nigerian actors encounter AI: aspirations of “responsible” and “inclusive” futures built on infrastructures that are themselves fragile and exclusionary.

Once past the reception bottleneck, the atmosphere inside was charged with a mix of excitement and urgency. Across the hall, young and ambitious Nigerian tech founders moved between stands and panels, carrying, as it were, the burden of repositioning Africa within futures that are already being shaped elsewhere by what the Afrofuturist thinker, Eshun, labels as the “futures industry” (2003). This is an industry that rarely focuses on the continent.

Philip exemplifies this passion and burden. His presence at the event, and more significantly the creation of Korin AI, signal an intense commitment to reclaim technological advancement through culturally grounded design. Rather than limiting himself to rhetorical appeals for African “inclusion” in AI, he adopts a praxis-oriented decolonial stance by attempting to build an African AI music generation platform from within the continent’s own linguistic and cultural realities. In so doing, this intervention resonates with decolonial thinkers such as Mignolo and Walsh (2018), for whom decoloniality is necessarily not only critique but praxis, rooted in the labor of creating alternatives from within the enduring structures of coloniality rather than waiting to be recognized by them.

If futures must be imagined before they are actualized, then Philip's own future consciousness around music production is revealing. As both a musician and a musicologist, he has studied how technological incursions have transformed music production over time. In our discussion, he narrated how he envisions “a future where you don’t have to actually play all of those Fruity Loops^[3]^ again.” Philip anticipates a move away from complex software interfaces toward natural language and simple hand gestures, using his hands to illustrate how AI systems could generate fully formed instrumentals and music that match what producers and artists imagine. This imagined future captures both the radical accessibility that could be enabled by GenAI and the potential erasure or reconfiguration of existing forms of musical labor, expertise, and collaboration within the Nigerian music ecosystem.

While these contradictory forces are evident across the study, the imaginative leap is very significant. It is Africanfuturist, because this future consciousness is marked by the insistence that Africans should not only inhabit the futures but actively imagine and shape its terms from within their own realities. Philip's projection, therefore, transcends technological optimism to become an anticipatory claim on what African musical futures could be. The force of such imagination matters, because decoloniality does not begin only with critique or refusal. It also begins with the ability to envision otherwise, to imagine new possibilities before they are institutionally or materially secured. In this instance, imagination, then, is not secondary to decolonial praxis but one of its first grounds. It opens the conceptual and affective space within which alternatives are desired and eventually actualized. Phillips therefore offers an Africanfuturist mode of consciousness, one in which technological possibility enables a way of reclaiming authorship over the shape and direction of African musical modernity.

While loud, upbeat Nigerian music pulsated in the background, Philip's phone buzzed incessantly as he toggled between coordinating with Solomon Ogunbowale, his co-founder and CTO who was attending another meeting elsewhere in Lagos, and corresponding with team members scattered across Nigeria. In Nigeria, remote working is seldom an indication of work flexibility; it is more often a strategy for surviving infrastructural challenges, such as unreliable electricity, unstable internet, hours of traffic, and the high cost of office space, especially for underfunded startups like Korin AI. As I was about to ask another question, he took a call from, as he would later reveal, a potential financier with whom he hoped to finalize a funding arrangement. Despite developing a visionary platform capable of reshaping the Nigerian music industry, he repeatedly emphasized that a lack of funds was stalling the company’s progress. This is not a problem that is unique to Philip. Disrupt Africa’s *African Tech Startups Funding Report* (2024) reveals that funding dropped by more than 50% between 2023 and 2024 (from US$2.4 billion to US$1.1 billion), marking a second consecutive year of decline.

In many ways, this is also what makes Korin AI and similar decolonial projects thoroughly Africanfuturist: their insistence on reimagining alternatives in the face of overwhelming scarcity. Drawing on his background, Philip frames Korin AI as a democratizing tool, designed to ease access to music production in Nigeria. Professional studio sessions remain prohibitively expensive for most Nigerians. “What takes two to three hundred dollars to record one song,” he explained, “with Korin AI, [takes] less than ten dollars. It’s fast. It’s affordable.” In this rendering, GenAI becomes both an enabler for efficiency and social equity. This ambition is grounded both in an entrepreneurial recognition of Nigeria’s infrastructural gaps and in his personal experience as a musician who has struggled with these limitations. This optimistic emphasis on speed and affordability, however, raises anxieties around authenticity and the aesthetic sensibilities that have historically characterized Nigerian music; a concern expressed by other interviewees.

Behind this technological formation lies a deeper cultural and political imperative. Philip repeatedly emphasized that African participation in the global AI race is not optional but existential. As he put it, one of Korin AI’s crucial priorities is cultural preservation. The conception of Korin AI thus transcends being a cheaper or faster alternative to produce Nigerian music, but as a preservationist and decolonial endeavor. According to him, if African sounds and languages are not actively encoded into AI systems, they risk either erasure or distorted return. This concern resonates strongly with both Africanfuturism and Decolonial AI and finds empirical support in the Mehta et al.(2024) study, which shows that African music corpora make up only about 0.2% of the more than 1 million hours of audio data used in AI music generation. One moment in particular foregrounds the affective force of this position. Recalling his encounter with early Western AI music labs, Philip described entering text in the expectation that the system would sing in Yorùbá, only to be met with something else entirely, “I was like, what’s this? That they are leaving Africa out completely in this AI race? Like now? Like it happened then?” The failure did not register for him as a minor technical glitch, but as the repetition of a familiar pattern in which Africa is once again left out of an emerging technological order. As he slowly paces through these rhetorical yet deeply charged questions, his brow tightened, his voice sharpened, and he leaned a little closer before letting out a disbelieving sigh. It was a look of decolonial vexation, marked by anger and weariness at a recurring pattern of exclusion, yet sustained by the conviction that things could still be otherwise. What surfaced in that moment was close to what Baldwin describes as the condition of critical consciousness among Black people: “to be relatively conscious is to be in a rage almost all the time” (Baldwin et al. 1961, p. 205).

Where others might simply accept such mis-renderings as inevitable, Philip engages them as part of a longer colonial pattern in which Africa is repeatedly positioned at the margins of technological modernity, either omitted altogether or included only in distorted, secondary form. His frustration is therefore borne out of the historical familiarity (consciously or otherwise) of the exclusion coloniality reproduces. In this regard, the founding of an African-owned and African-trained AI company can be understood in light of Mignolo’s (2011) “epistemic disobedience,” which in this instance is a refusal to accept Africa as a passive consumer of imported algorithmic systems, and an insistence on participating in the definition of the data and aesthetics through which AI is imagined, designed, and developed. Korin AI thus emerges both as a technical and as an onto-epistemic intervention, seeking to shift Africa from being reduced to raw materials or data for external computation toward claiming agency as a computational center.

This epistemic stance is equally visible in Philip's refusal of deficit narratives about Africa’s place in technological history. “I don’t want to use the words, ‘we are behind’,” he insisted. “We are not behind. Just part. We are part in the journey.” Though seemingly simple, this formulation unsettles what Sprengel (2023) describes as the logic of “imperial lag,” the colonial temporality through which Africa is cast as perpetually late, catching up, or outside the proper time of modernity. This deconstruction of temporality and modernity is at the heart of decoloniality and Africanfuturism. Against this linear ordering of time, Philip frames African AI-making as coeval with, rather than subordinate to, other technological futures. The point is not that Africa will someday arrive at a destination already mapped up elsewhere, but that it is already present within the journey, even if on unequal terms. His language therefore performs an important temporal correction by rejecting the idea that African participation in AI must always be derivative or belated, and instead claims simultaneity, presence, and agency in the making of technological futures.

### Data, infrastructures, and the limits of decoloniality

Nevertheless, the structural barriers are real, and Philip is far from naïve about the challenges. His optimism is consistently tempered by the material constraints within which Korin AI operates. Like several other interviewees, he repeatedly returns to one central bottleneck: data. AI systems are only as powerful as the datasets that train them, and in this regard, Korin AI operates at a stark disadvantage. Philip contrasts Korin AI’s situation with that of GenAI companies based in Global North, such as Suno or Udio, which in his view appear to have comparatively easier access to large-scale catalogs and institutional archives, even if ongoing legal disputes suggest that much of this training has drawn on undisclosed and copyrighted corpora. By contrast, he notes that a Nigerian start-up seeking comparable datasets often has to formally request access from Global North-based technology companies that are under no obligation to respond, let alone grant such access.

One more central contradiction in the encounter between GenAI and Nigerian music becomes visible here. The problems of compiling an adequately large and representative African-music corpus are indicative of a broader Nigerian and African data landscape in which fragmented and low-quality datasets still pose serious obstacles to advances in computing and AI (Effoduh et al. 2024; Katende 2025). As Ndlovu-Gatsheni notes, African futures are invented “under and within conditions of global coloniality” (2014), including the infrastructures of platforms and AI systems. While these digital technologies foster visibility and circulation, they also re-enact exclusion and displacement, particularly for those on the wrong side of infrastructural divides.

Korin AI’s founders described their approach to data acquisition as one intended to differ from the more extractive practices associated with some global competitors. They reveal that the company works directly with Nigerian producers and artists, compensating them for access to archival materials and, in some cases, commissioning new recordings specifically for model training. This is a costly and slow approach for a small start-up dependent on intermittent funding, but one that is more consistent with decolonial commitments to consent, compensation, and value redistribution. However, even if Korin AI is attempting to build a more ethically sourced corpus, the servers, software frameworks, and computational architectures on which its models run remain tethered to infrastructures in Global North. Decolonial AI, in this instance, is both aspirational and compromised, an attempt to provincialize and redirect AI from within infrastructures that still bear the imprint of global coloniality.

### The representation of languages and genres

Similar tensions are present in Korin AI’s language support approach. The platform currently prioritizes Nigeria’s dominant languages (Pidgin, Yorùbá, Hausa, Ígbò) and English, with plans to expand. The ambition is to eventually support over one hundred African languages. However, the criteria for which languages are supported first are shaped by the economics of demand and platform affordances, rather than by a logic of cultural value and decolonial ethics of reparative attention to marginalized African languages. This is where decolonial commitments run into the profit imperative of techno-capitalism. As Philip acknowledges, Korin AI must “look at the demand, the need…We can’t cover all the languages first.”

Therefore, while Korin AI positions itself against Western-centric models by foregrounding African languages, it simultaneously reproduces internal hierarchies of visibility in which already dominant Nigerian languages are further consolidated, while hundreds of minority languages remain structurally peripheral. The category “African language” thus risks functioning as a homogenizing abstraction that masks the uneven terms on which inclusion takes place. In practice, some languages are likely to be deeply integrated into the model’s capacities, refined through repeated use and optimization, while others may be added as symbolic gestures of presence, included, as Philip puts it, “just to have it there.” What appears at the level of discourse as broad linguistic inclusion therefore becomes, at the level of implementation, a differentiated and selective politics of representation.

The contradictions of decolonial AI are more glaring here. The effort to correct one axis of exclusion can easily (de)generate new internal margins, because the project must still operate through logics of scale, demand, technical feasibility, and market prioritization. Korin AI’s language strategy thus reveals how decolonial projects can become sites of selection as much as resistance, forced to sort, rank, and defer even as they seek to widen the field of inclusion. In this regard, the platform does not escape the turbulence of coloniality; it navigates it, and in navigating it, reproduces some of its familiar pressures in altered forms. The problem is not simply that some languages are left for later, if they are reached at all, but that “later” itself becomes a politically charged temporal category, one that quietly distinguishes between languages deemed central to the future and those rendered supplementary to it.

Solomon Ogunbowale, the second co-founder and CTO of Korin AI, provides a detailed explanation of how these hierarchies are expressed through language. In his assessment, Nigerian Pidgin currently yields some of the best outputs, followed by Hausa and then Ígbò, while Yorùbá is paradoxically more challenging. He attributes this difficulty to the tonal structure of the language, in which pitch is not only expressive but, more importantly, meaning bearing, such that a single orthographic form can shift semantically depending on tone. This is consistent with broader scholarship on Yorùbá language technologies, which identifies tone handling as a major challenge in speech synthesis, speech recognition, and NLP, because meaning depends so heavily on pitch (Van Niekerk and Barnard 2014; Jimoh et al. 2025; Olusanya 2026). Korin AI’s difficulty with Yorùbá therefore does not reflect a local anomaly so much as a wider problem in language technologies, which are developed within uneven data and computational systems that tend to privilege languages whose semantic and phonological structures are easier to standardize. This exemplifies how language-specific computational constraints compound existing inequalities in decolonial inclusion. Even within Nigeria, some languages are computationally easier to render than others, and “African-trained,” as in the case of Korin AI, becomes a contested category in which tonal and minority languages risk being pushed further to the margins, because they pose greater challenges for existing architectures or promise weaker immediate commercial returns.

A parallel hierarchy operates at the genre level. While Korin AI aspires to reflect the breadth of African musical traditions, popular and exportable forms, such as Afrobeats, Afropop, and Afro-R&B, are far more likely to be prioritized and trained than less globally visible genres like Waka, Sakara, Ikwokirikwo, or Dadakuada. This is not simply a matter of aesthetic preference; it reflects how decolonial projects, even when explicitly oriented toward African representation, remain entangled in infrastructures and market logics that reward familiarity and metric performance. Genres already legible and “streamable” as “African music” within dominant digital systems are more easily absorbed into AI training systems, while others remain peripheral or absent. In this way, efforts to decentralize Western hegemony do not escape the architectures through which the existing hegemony continues to operate. Instead, they often reproduce internal hierarchies by privileging dominant genres and forms, leaving others to be addressed later, if at all. GenAI thus participates in yet another round of aesthetic reconfiguration in Nigerian music, further amplifying certain histories and sonic textures while muting others. An attending consequence is that some genres would come to stand as the default sound of Nigerian/African musical futurity, while others remain at the margins of that imagined future.

## Conclusion

Nigeria’s music industry is globally visible yet internally heterogeneous, shaped by multiple traditions even as Afrobeats has come to dominate its international perception. At the same time, Nigerian musicians continue to work within deep infrastructural, legal, and socio-economic constraints that make music production difficult and expensive. Accessible technologies have long been central to navigating these conditions, and GenAI now appears as the latest layer in this history of adaptation. Unlike in many Global North contexts, where lawsuits, artist boycotts, algorithmic aversion, and public controversies have framed GenAI primarily as a site of crisis, the Nigerian context appears less overtly turbulent. This relative quiet does not indicate lower stakes. Rather, it reflects how GenAI is taking shape within an industry already structured by infrastructural scarcity, weak legal enforcement, and chronic precarity.

The significance of Korin AI is not in having resolved these contradictory conditions, but in revealing what AI-building in the Global South looks like when it must emerge from within the ruins of coloniality/modernity. As we have shown, Korin AI should be understood neither as evidence of a fully realized decolonial alternative nor simply as a case of technological dependency. It is better read as a compromised but meaningful project of future-making, an attempt to reshape the terms of AI-mediated music production in Nigeria and, by extension, Africa, while remaining tethered to the uneven systems of data, computation, law, and capital through which AI currently operates. Its importance, then, is not that it escapes coloniality, but that it makes visible the contradictions involved in trying to redirect AI from within it.

At present, Korin AI appears to be among the earliest, and perhaps the most clearly articulated, African-founded attempt to build a dedicated generative music platform trained around African musical data. While adjacent AI-audio ventures may exist, we found little evidence of a directly comparable Africa-based music-generation platform documented in public sources.

The wider fieldwork also reveals that there are no absolute positions on AI’s intersection with Nigerian music. What emerges instead is a spectrum of responses, ranging from uncertainty and caution to experimentation, strategic leverage, and the hope that AI might lower barriers to production or extend existing currents of musical innovation. However, some of the strongest resistance centers on GenAI’s perceived inability to reproduce the socio-cultural sensibilities that characterize Nigerian music, and its inability to inhabit the human-centered performance practices through which that music is made and received. Korin AI embodies these larger tensions as its development is rooted in this atmosphere of ambivalence, which it must also work through it. Its acceptance and eventual success, if these are to come, will depend on both its technical capacities and more importantly, how convincingly it responds to the doubts, resistances, and aspirations that already shape Nigerian musicians’ encounters with GenAI.

In both genre and technology, the adaptability of Nigerian music has long been recognized as innovative, creative, and survivalist.^3^ The question, then, is whether the Nigerian music-tech sector can build systems that do not only reproduce generic models of “African” sound, but instead encode, support, and extend the specific musical intelligences through which Nigerian music has historically moved between the local and the global. Examined through Africanfuturist and Decolonial AI lenses, Korin AI foregrounds a central tension. On one level, it embodies decolonial and Africanfuturist aspiration by training models on African data, foregrounding local languages and genres, and insisting that African sounds should not remain absent from AI’s musical futures. On another level, it remains entangled in the infrastructures of global coloniality, dependent on cloud systems located outside Africa, constrained by unequal access to data, and compelled to make strategic decisions about which languages and musical forms are worth encoding first. What emerges, then, is not a simple story of inclusion or exclusion, but a contradictory process in which efforts to build alternative futures also reproduce new internal hierarchies and exclusions.

The case of Korin AI therefore complicates celebratory narratives of AI “inclusion” by showing that decolonial aspirations are always mediated by the material and institutional realities through which they must pass. At the same time, it pushes back against accounts that reduce AI in the Global South to passive adoption or dependency. The insights presented here show that Nigerian actors are actively struggling over what AI will mean, whom it will serve, and which musical futures it will make possible. As we have argued throughout, this Africanfuturist and decolonial development of GenAI in Nigeria is fraught with contradictions and structural constraints. Regardless, because it attempts to build from within those constraints, it stakes a meaningful claim toward a more just and preferable African future, even if that future is, for now, more anticipated than fully realized. Korin AI, in this regard, demonstrates that Africanfuturist and decolonial projects in music AI are not abstracted alternatives standing outside modernity/coloniality, but necessary and contested efforts to reorganize musical futures from within its enduring structures.

## Data Availability

No datasets were generated or analysed during the current study.

## Footnotes

1 A related problem is lack of transparency when it comes to training data. A recent study of 27 AI models for music generation found that only 5 of the models declared which training datasets were used in training the AI model. Only 11 of the 27 models declared which AI techniques are used to generate music (Wilson et al 2025). This of course makes it very difficult to evaluate the fairness with which AI models represent different musical styles and cultures.

2 Moreover, “mode collapse” and convergent prompting have been shown to flatten stylistic diversity, with multiple studies documenting reductions in novelty and variation in the context of GenAI-assisted music production (Zhou and Lee 2024; Holzner et al. 2025).

3 Okpeki and Oje (2019, p. 189–91) trace the transition from the live-band era (1940s–1980s), dominated by artists such as Fela Anikulapo-Kuti, Ebenezer Obey, and Victor Olaiya, to the digital studio age, where synthesizers and electronic keyboards replaced live instrumentation. They show how the latter facilitated precision and reduced production costs but also deepened the individualization of music-making.
